# Neurocysticercosis by *Taenia* sp. in a female alpaca (*Vicugna pacos*) from Germany

**DOI:** 10.1186/s12917-026-05502-y

**Published:** 2026-04-21

**Authors:** Martin Dembowski, Lilli Bittner-Schwerda, Kristin Pütsch, Zaida Rentería-Solís, Shenja Loderstedt, Ingmar Kiefer, Alexander Starke, Christiane Helm, Florian Hansmann

**Affiliations:** 1 Institute of Veterinary Pathology, Faculty of Veterinary Medicine, Leipzig University, Leipzig, Germany; 2 Clinic for Ruminants and Swine, Faculty of Veterinary Medicine, Leipzig University, Leipzig, Germany; 3 Institute of Parasitology, Faculty of Veterinary Medicine, Leipzig University, Leipzig, Germany; 4 Department for Small Animals, Faculty of Veterinary Medicine, Leipzig University, Leipzig, Germany

**Keywords:** Neurocysticercosis, Alpaca, Germany, *Taenia* sp.

## Abstract

**Background:**

Neurocysticercosis, caused by *Taenia* sp., represents one of the most important parasitic infections of the central nervous system (CNS) globally, with low but increasing numbers of documented cases in both animals and humans in Europe. *Taenia* sp, has been documented as etiological agent of disease in several domestic and wildlife animals worldwide as well as in humans. However, it has not yet been reported in New World Camelids (NWC).

**Case presentation:**

A 7-year-old, female alpaca was presented due to progressive neurological signs including ataxia and progressive weakness of the hindlimbs. With the aid of clinical examination and imaging procedures the presence of multifocal lesions in the brain and spinal cord was demonstrated, and *Taenia* sp. were suggested as causative agent via serology. After a treatment attempt the animal was euthanized and submitted for necropsy due to the deterioration of clinical signs. Macroscopic, histopathological, histochemical and immunohistochemical *postmortem* investigations confirmed multifocal mineralisations with surrounding fibrosis, myelin sheath dilation and adjacent astrogliosis and microgliosis.

**Conclusions:**

Neurocysticercosis causes severe, often lethal and diagnostically challenging infections, like in the present case. Therefore, this disease should be included in the list of differential diagnosis in wildlife and domestic animals including alpacas with unspecific neurological signs.

## Background

The alpaca, a member of the new world camelids (NWC), is experiencing a surge in popularity in Germany, which is concurrently increasing the necessity for veterinary healthcare services for NWC [1, 2]. The susceptibility of alpacas to a multitude of infectious agents continues to present a diagnostic challenge for veterinarians [1, 3]. Especially gastrointestinal diseases frequently caused by endoparasites are a prevalent occurrence in alpacas [4]. In addition, neurological diseases, caused by infectious agents or traumatic insults are common clinical presentations in NWC [4, 5]. Neurological diseases caused by the “meningeal worm” *Parelaphostrongylus tenuis* are quite common in North America [6], whereas parasitic neurological diseases are relatively rare in Europe as highlighted in a report of *Baylisascaris procyonis* in the central nervous system (CNS) of an alpaca [4]. Animals with parasitic infections in the CNS show, among others, mainly neurological deficits including ataxia, hind limb weakness, circling and blindness [5]. The family of Taeniidae is a highly relevant member of the Plathelmintes in veterinary medicine, as it also includes the genus Echinococcus apart from the genus *Taenia*. Both genera are distributed worldwide and are known to cause severe, potentially lethal diseases [6]. All *Taenia* sp. undergo a diheteroxenic development cycle. The adult forms are naturally harboured in the small intestine of the definitive host, humans or carnivores, and are low pathogenic. The released infectious eggs are ingested by the intermediate host, mainly herbivores, and the emerging oncosphere traverse the intestinal epithelium and lodge in tissues, where they differentiate into the cysticercus, depending on the subspecies in the musculature, subcutaneous tissue and body cavity but rarely also into other parts of the body including the central nervous system (CNS) [7, 8]. These cysticerci establishing in the brain cause the so called neurocysticercosis [7, 8]. Clinical disease caused by cysticerci is a significant cause of epilepsy in humans and a major human public health concern in regions where the genus *Taenia* is endemic [9–12]. Due to its diverse range of clinical manifestations, cysticercosis in the CNS remains particularly challenging, necessitating a variety of diagnostic procedures, including neuroimaging, serological and molecular biological examinations [13]. However, there have been no documented case of neurocysticercosis in alpacas to date. This case report describes the clinical presentation, the diagnostic approach and the gross and histopathological workup of an alpaca with neurological signs caused by neurocysticerosis.

### Case presentation

A 7-year-old female alpaca was presented at the Clinic for Ruminants at the Faculty of Veterinary medicine in Leipzig, Germany. For a week prior to presentation, the patient, from a small-scale hobby holding, with one more female alpaca, had been demonstrating an unsteady gait and difficulty standing up and was treated with non-steroidal anti-inflammatory drugs.

At presentation the diagnostic measures included a clinical and neurological examination [14–17], blood analysis and examination of cerebrospinal fluid, coprological examination as well as magnetic resonance imaging (MRI) and computed tomography (CT).

The clinical examination revealed: reduced demeanor, increased heart rate of 80 beats per minute with a regular arrythmia, reduced rectal temperature 37.1 °C, increased episcleral vessel filling, reddened oral mucosa. Upon arrival the animal was able to stand with assistance for a short time with a pronounced weakness in the hindlimbs. The head showed an intermittent shaking movement. In the neurological examination the cranial nerve functions (N. opticus, N. intermediofacialis, N. accessories, N. occulomotorius, N. glossopharyngeus, N. hypoglossus, N. trigeminus) were unremarkable, and reflexes (panniculus reflex, flexor reflex) at all four limbs were slightly exaggerated. Hematology and blood chemistry showed a slight eosinophilia (3.6 G/l; reference range: 0.8–3.4 G/l) and hypocalcaemia (1.98 mmol/l; reference range: 2.1–2.5 mmol/l) [18]. Selenium was within reference range (196 µg/l; reference range: 114–282 µg/l [19]. The analysis of the cerebrospinal fluid was unremarkable. In the coprological examination a low amount of Capillaria and Nematodirus eggs was detected.

**Fig. 1:**
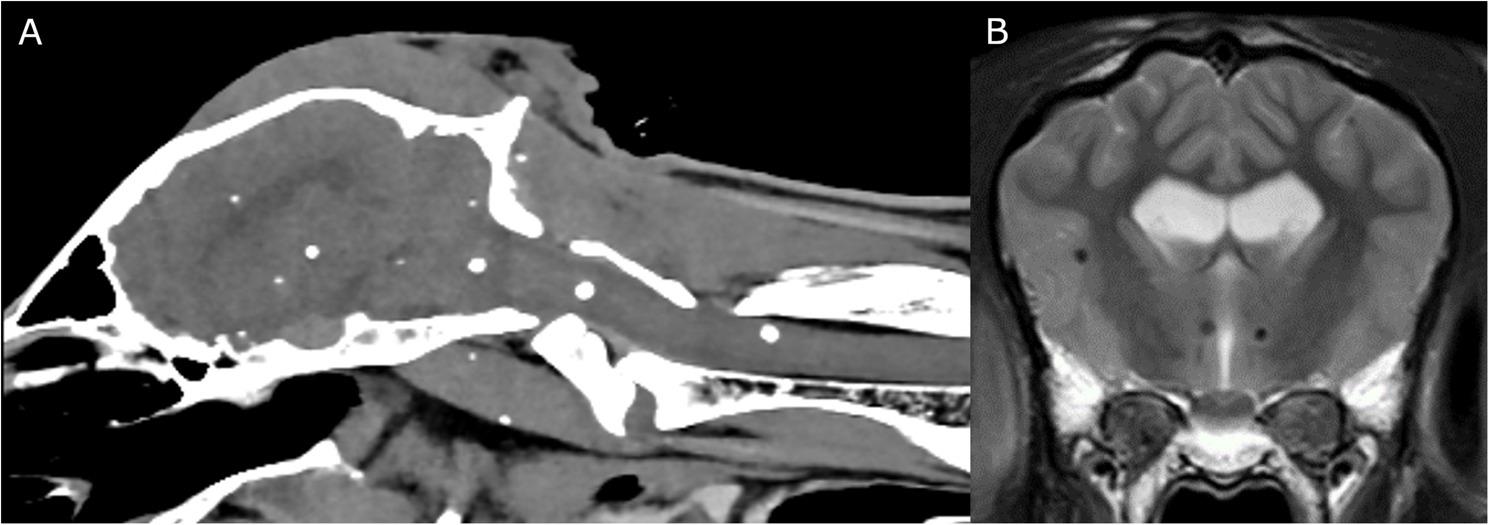
Computed tomography (CT) sagittal head and cervical spine up to vertebral body C2. Multiple mineral-dense hyperdense structures are present in the brain and spinal cord as well as the adjacent muscles (**A**). Transverse T2 magnetic resonance imaging (MRI) of the cerebral cortex and thalamus revealed multifocal hypointense lesions (**B**)

In consequence a CT and MRI were planned. The MRI revealed multiple well-defined, spherical, T2-, FLAIR-, and T1-hypointense lesions of varying sizes (about 5 mm in diameter) in the parenchyma of the cerebrum, cerebellum, and brainstem, as well as within the cervical spinal cord. These lesions appeared hyperdense on the CT scan. The lesions were interpreted as multifocal intra-axial mineralisations (Fig. 1) suggestive of parasitic cysts. Additionally, a mild protrusion of the intervertebral disc between C2 and C3 with mild compression of the spinal cord was evident.

*Taenia* sp. specific antibodies were identified in the blood serum using a commercial kit (VetLine Taenia, NovaTec Immunodiagnositca, Dietzenbach, Germany). The suspected diagnosis was neurocystercosis caused by *Taenia *sp. Following the human medicine guidelines for the treatment of neurocysticercosis [20, 21], the following therapeutic attempt was performed: Initially the alpaca received parenteral fluids (0.9% saline, 2–4 mg/kg bodyweight (BW) per hour), antibiotics (Amoxicillin-Trihydrat; Betamox^®^; 7 mg/kg BW for 18 days) and non-steroidal anti-inflammatory drugs (Meloxicam 20 mg; Metacam^®^ 0.5 mg/kg BW). Furthermore, three times daily physiotherapy was done, by assisting the animal standing and active mobilisation of the joints. Based on the results of the diagnostic imaging and parasitological examinations, antiparasitic treatment was initiated three weeks after admission, consisting of 470 mg of fenbendazole (Panacur^®^ 187.5 mg/g) administered orally once daily for 6 weeks. Clinical presentation after arrival deteriorated and the animal became recumbent being not able to stand even with assistance over a period of six days. Within one week after commencing oral antiparasitic treatment, standing ability improved progressively and the alpaca was able to walk for a short distance. However, after this short phase of improvement, the condition of the animal deteriorated and the alpaca became recumbent again. The owner restrained from further therapy, and after nine weeks of hospitalization the alpaca was euthanized by intravenous administration of 0.4 mg/kg xylazine, followed five minutes later by 5 mg/kg ketamine intravenously. Once adequate sedation was achieved, 15 ml T61 was intravenously administered.

**Fig. 2:**
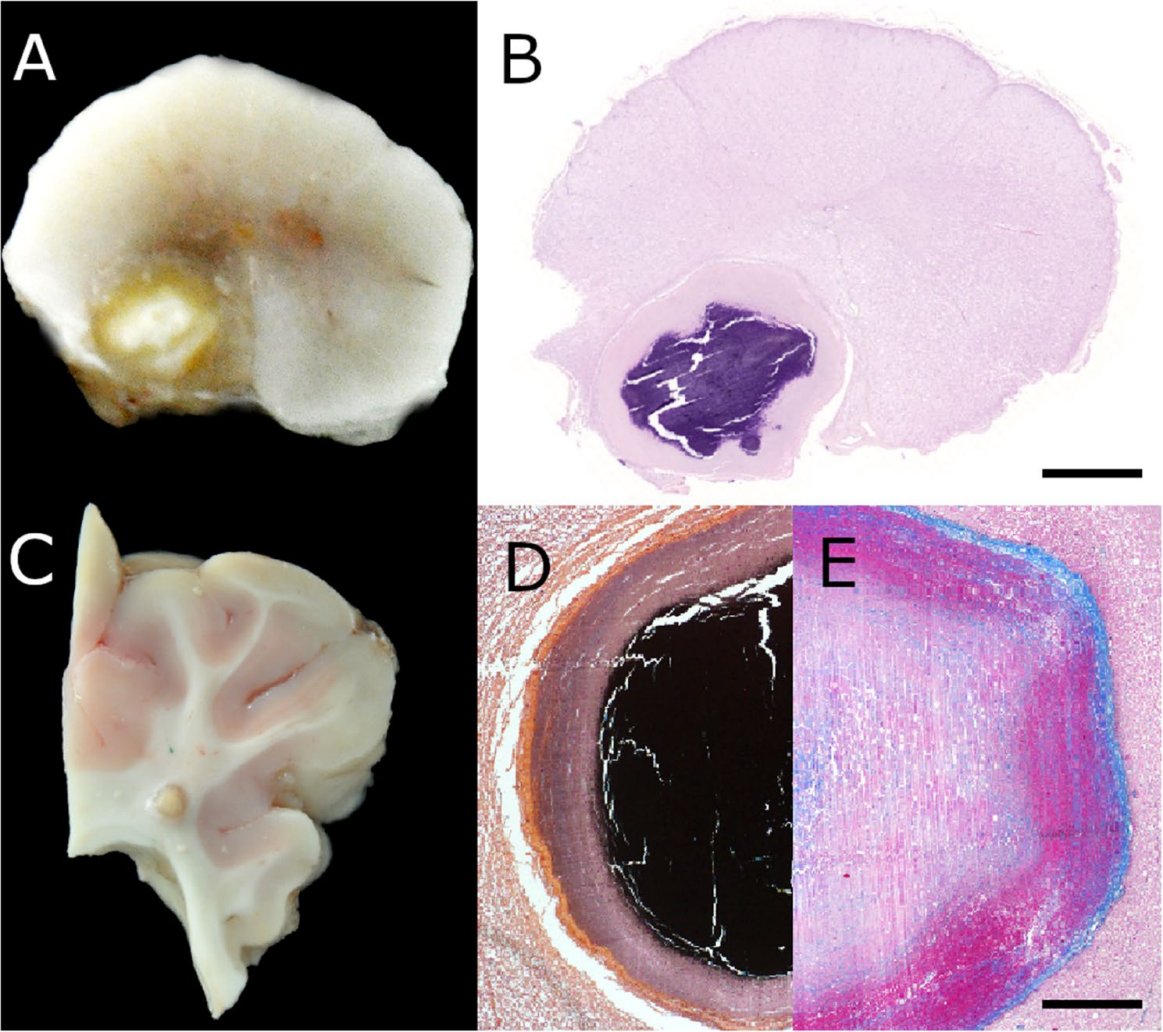
Spinal cord at the level of the second cervical vertebra (**A**, **B**, H&E staining) and cerebrum, lobus parietalis (**C**). A 4 mm in diameter large, hard nodule was detected. The nodules consisted of calcified material as identified by von Kossa-silver impregnation (**D**) which was surrounded by fibrosis (**E**, Azan staining). Bars: B: 1 mm, D/E: 500 µm

At gross examination multiple, pale yellow, spherical and rigid structures measuring up to 5 mm in diameter in the cerebrum, the cerebellum and the spinal cord as well as a mild protrusion of the intervertebral disc between C2 and C3 were identified (Fig. 2A).

**Fig. 3:**
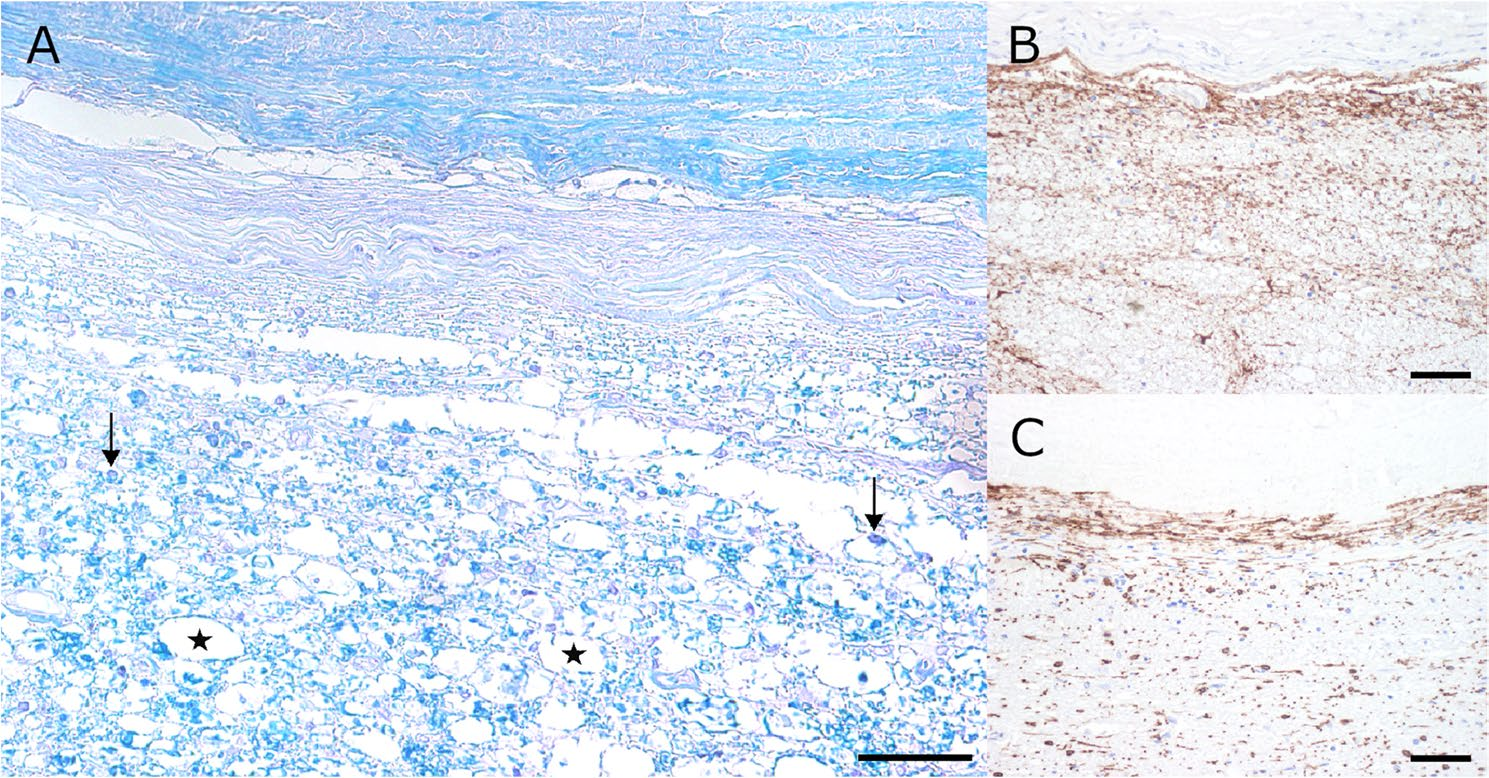
Spinal cord at the level of the sixth cervical vertebra. **A** Luxol fast blue staining was used for visualization of dilated myelin sheaths ($\ast$) and myelinophages ($\downarrow$). Immunohistochemistry targeting glial fibrillary acidic protein (GFAP) identified an astrogliosis demarcating the lesions (**B**). In addition, a locally extensive microgliosis was detected by ionized calcium-binding adaptor molecule 1 (Iba-1, **C**). Bars: 50 µm

Tissue samples for histological, histochemical and immunohistochemical analysis were collected from brain, spinal cord, peripheral nerves, lung, liver, spleen, kidneys, heart, eyes, thyroid, and others, fixed in 10% neutral buffered formalin overnight followed by dehydration and embedding in paraffin wax. Sections were cut at 2-µm thickness and stained with hematoxylin and eosin (HE). Histochemical special stains and immunohistochemistry were performed as previously described [22, 23]. Histopathological lesions were restricted to the CNS and included multiple spherical structures within brain and spinal cord (Fig. 2B). The spherical structures consisted of calcified material as identified by von Kossa-silver impregnation (Fig. 2C). The lesions were surrounded by a rim of fibrosis as visualized by Azan special stain (Fig. 2D). The adjacent neuroparenchyma displayed multiple dilated myelin sheaths, swollen axons (spheroids) and multifocal myelinophagia (Fig. 3A). Employing immunohistochemistry an astrogliosis and microgliosis demarcating the mineralisations was detected (Fig. 3B, C).

Identification of the *Taenia* sp. was attempted by conducting a taeniid-specific PCR assay aimed to amplify a fragment (373 bp) of the 12 S mitochondrial rRNA gene [24, 25]. Briefly, DNA extraction was conducted using the DNeasy Blood and Tissue Kit (Qiagen, Hilden, Germany) following the manufacturer’s instructions. Each PCR reaction consisted on 2.5 µl of DreamTaq™ Green Buffer (10x, Thermo Fisher Scientific, Dreieich, Germany), 0.2 µM of each deoxynucleoside triphosphate (dntp), 1U of DreamTaq Polymerase (Thermo Fisher Scientific), 3 µl of template (purified DNA). Negative and positive controls were added to the PCR assay and consisted on DNA/nuclease-free water and genomic DNA from a sample previously identified as *T. martis* (Reference for positive control: [26]). PCR cycle conditions were as follows: initial denaturation at 94 °C for 2 min (min), 35 cycles of 94 °C for 30 s (s), 58 °C for 30s, and 72 °C for 1 min, a final amplification cycle of 72 °C for 5 min was added to the cycle. All PCR reactions were run in a Biometra Tadvanced^®^ thermal cycler (Analytik Jena, Jena, Germany). However, it was not possible to obtain PCR products of sufficient quality and sequencing of such PCR amplicons was unsuccessful. This outcome could likely be due to the calcification of the lesions.

## Discussion and conclusions

This study presents a case of an alpaca exhibiting progressive neurological signs due to multiple lesions in the CNS most likely caused by *Taenia* sp., a condition known as neurocysticercosis. In the present case, mineralisation within the CNS including brain and spinal cord in combination with a seropositivity of *Tania sp.* were detected, while no viable stages of *Taenia* metacestodes were observed by histopathology. In addition, PCR failed to detect *Taenia* sp. specific nucleic acid sequences within lesioned CNS material. However, serologically *Taenia* sp. specific antibodies were detected, although serology is often false negative [9]. Therefore, other causes including a migration of larvae from *Toxocara* sp. or *Baylisascaris* sp. should be taken into consideration. However, migrating larvae of these parasite species are frequently associated with marked eosinophilic inflammation [27–30]. Although mineralised lesions occur infrequently, lesions overall appear much smaller than those observed in the present case [27, 30]. Moreover, one case report details a cerebral infection in an alpaca caused by *Elaphostrongylus cervi* [31]. *Parelastrongylus* sp. can cause comparable brain lesions by an aberrant larval migration, however larvae themselves measure only 90 μm in diameter [31, 32]. This differential diagnosis is very unlikely since the occurrence of this parasite has not been reported in Europe yet. Other diseases of the CNS most likely causing focal lesions, include bacterial or viral encephalitis [5]. However, lesions caused by bacteria are typically associated with a pronounced neutrophilic or mononuclear (viruses) inflammatory reaction which may be associated with mineralisation. Finally, neoplastic or degenerative processes may theoretically account for focal lesions but would rarely explain a similar, multifocal distribution. Summarized, the distribution, morphology and mineralised nature of the lesions are consistent with a *Taenia* sp. infection.

The most important *Taenia* sp. in humans are *T. solium*, *T. saginata* and *T. asiatica*, whereas the latter is mainly distributed in the South and East of Asia [33–35]. In central Europe, in addition to the taeniid species described in humans, *T. hydatigena*,* T. pisiformis*,* T. ovis*,* T. krabbei*,* T. multiceps*,* T. serialis*,* T. crassiceps*,* T. polyacantha* and *Hydatigera taeniaeformis -* as well as *T. martis* and *Versteria mustelae* are also found [36]. *Taenia* sp. generally have a wide range of intermediate hosts. Cattle, sheep, goats, roe deer, buffalo and yaks serve as intermediate hosts for *T. saginata*,* T. hydatigena*,* T. ovis*,* T. krabbei* and *T*. *multiceps*. These *Taenia* sp. should therefore be considered as potential causes in the present case [36].

The seroprevalence of *T. saginata* in cattle in Germany, as determined by ELISA, is 8.83% [37]. For *T. hydatigena* a prevalence of 14.6% was found in lambs from Sardinia [38] and nearly 30% in Greece [39]. During slaughter inspections, infection with *T. multiceps* was detected in about one-fifth of the sheep examined in Italy and Turkey [40]. Despite case studies, no prevalence data are available for other *Taenia* sp., particularly in Germany.

The alpaca in question comes from a small herd, that includes one other alpaca. Therefore, contact with a final host appears possible and even likely. In Germany, foxes in particular, but also dogs and wolfs, should be considerated in Germany [41].

In foxes, *Taenia* sp. were detected in approximately one-third of the animals examined [42]. However, recent data are lacking. In 1992 and 1997, *T. crassiceps*,* T. polyacantha*,* H. taeniaeformis*,* T. serialis*,* T. pisiformis* and *T. martis* were the most commonly detected species [43, 44]. In dogs, however, the prevalence was very low [45]; while older studies considered *T. hydatigena* and *T. pisiformis* to be the most common species in Europe [36], *T. crassiceps*,* T. martis*,* T. serialis*,* T. polyacantha*,* H. taeniaeformis* and *T. pisiformis* were also detected [46]. Parasitological examination of wolves revealed the presence of *Taenia* sp. in approximately 50% of the animals, with *T. hydatigena* being the most common species detected, although *T. krabbei* was also found [47]. In the acute phase, larval migration causes tissue necrosis accompanied by haemorrhage, followed by inflammatory and reparative processes including granulation tissue and fibrous capsule formation [36]. The calcifications observed in the present case were most likely of a dystrophic nature and secondary to cellular degeneration and necrosis, moreover multiple studies indicate that chronic lesions tend to form fibrocalcified nodules upon parasite elimination [10, 48–50]. The lesions also caused compression and damage to adjacent neuronal and axonal structures, leading to a disruption in signal transmission and consequently to the observed neurological signs in the present case.

Parasites within the brain parenchyma tend to die even without treatment [51]. However, parasites located outside the brain parenchyma (extraparenchymal neurocysticercosis), for example in the ventricles or the subarachnoid space, can be harder to diagnose [51]. They can also result in a deteriorated immune response and persist for a long period of time [51]. The role of the antiparasitic treatment remains unclear in this case. It is possible, that the treatment contributed or caused the death of the parasites leading to the initial improvement of the clinical signs after the start of the therapy. However, the immune response itself may also have resulted in the death of the parasites. Secondary inflammatory and degenerative changes likely contributed to the progressive clinical signs observed later in this case, as only multifocal calcified lesions surrounded by fibrosis were detected. The mild protrusion of the intervertebral disc was, most likely did not contribute significantly to the observed neurological sign due to its minor extent. Given the presence of multiple lesions in various regions, no consistent neurological pattern was discernible, which was likely responsible for nonspecific clinical presentation observed in this case.

The observed lesions of the neurocysticercosis in this case are largely comparable to those documented in humans, where number and location of the lesions, as well as the immunological reaction, is key to the clinical manifestation [20, 49, 52]. It has been reported that various animals serve as accidental intermediate hosts for *Taenia* sp., underscoring the broad host range and the opportunistic nature of these parasites. In the literature disseminated infections with *T. crassiceps* in dogs, cats, lemurs, squirrels, and Senegal bushbabies, frequently occurring in animals with compromised immune system, suggesting that immunosuppression may facilitate larval colonization [53–55]. However, an immunosuppression is not a prerequisite for developing neurocysticercosis [9]. In the present case, no morphological changes indicating an immune suppression were detected.

Neurocysticercosis has been reported in a dog infected with *T. solium* [56] and in a cat with *T. crassiceps* [54], both of which exhibited nonspecific neurological signs. In the dog, calcified myocardial nodules comparable to those observed in the present case were reported. It is noteworthy that parasitic structures can usually be detected in the lesions of these animals. However, it should be noted that their presence is not consistent in all cases.

In intermediate hosts, cysticerci are not limited to muscle or peritoneal tissue. Rather, they can settle in various organs including the CNS, where lesions may calcify after the parasite dies. The clinically detectable neurological deficits are attributable to the damaged the CNS tissue [36]. The neurotropic *T. multiceps* provides a classical example in sheep, producing CNS lesions causing neurological signs [57]. In experimental studies, *T. solium* was directly implanted into the CNS of pigs to induce changes comparable to human neurocysticercosis, where partially calcified nodules similar to those in the present case were observed [58]. Moreover, similar calcified lesions to the ones observed in the present case were also found in the CNS in pigs naturally infected with *T. solium* [35].

While *T. solium*,* T. saginata* and *T. asiatica* are well-recognized causes of human infection, particularly neurocysticercosis [59]; human infections with *T. crassiceps*,* T. multiceps* and *T. serialis* are rare [60]. To date, no zoonotic potential has been documented for other *Taenia* sp. discussed in this report.

Summarized, the susceptibility of NWC to various pathogens underlines the importance of adequate health care of these species. The present case underscores the necessity of including neurocysticercosis in the list of differential diagnosis of alpacas suffering from neurological signs.

## Data Availability

All data obtained or analysed as part of this study are included in this article.

## Abbreviations

CT: Computed tomography
CNS: Central nervous system
DNA: Deoxyribonucleic acid
HE: Hematoxylin and eosin
MRI: Magnetic resonance imaging
PCR: Polymerase chain reaction
N: Nervus
NWC: New world camelid
T: Taenia
BW: Body weight
